# Genome‐Wide Associations of Cognitive Domains and their Correlation with Polygenic Risk Scores for Antidepressant Response in Late‐Life Depression

**DOI:** 10.1002/alz.095772

**Published:** 2025-01-09

**Authors:** Samar Elsheikh, Victoria Marshe, Malgorzata Maciukiewicz, Farhana Islam, Xiaoyu Men, Vanessa SM Gonçalves, Daniel Felsky, James L. Kennedy, Benoit H. Mulsant, Charles F. Reynolds, Eric J. Lenze, Daniel Mueller

**Affiliations:** ^1^ Centre for Addiction and Mental Health, Toronto, ON Canada; ^2^ Colombia University, New York, NY USA; ^3^ University of Zurich, Zurich, Zurich Swaziland; ^4^ Center for Addiction and Mental Health, Toronto, ON Canada; ^5^ Krembil Centre for Neuroinformatics, Centre for Addiction and Mental Health, Toronto, ON Canada; ^6^ University of Toronto, Toronto, ON Canada; ^7^ Department of Psychiatry, Temerty Faculty of Medicine, University of Toronto, Toronto, ON Canada; ^8^ Campbell Family Mental Health Research Institute, Centre for Addiction and Mental Health, Toronto, ON Canada; ^9^ Adult Neurodevelopment and Geriatric Psychiatry Division, CAMH, Toronto, ON Canada; ^10^ University of Pittsburgh Medical Center, Pittsburgh, PA USA; ^11^ Washington University School of Medicine, St. Louis, MO USA; ^12^ 250 College St, Toronto, ON Canada

## Abstract

**Background:**

Late‐life depression (LLD) often coincides with cognitive decline, impacting antidepressant treatment outcomes. Investigating the genetic profile of cognitive function and its association with antidepressant response in individuals with LLD is crucial.

**Method:**

In the Incomplete Response in Late‐Life Depression: Getting to Remission (IRL‐GRey) study, 307 older adults with major depressive disorder underwent 12‐week venlafaxine treatment. A genome‐wide association study (GWAS) of five cognitive domains using the Repeatable Battery for the Assessment of Neuropsychological Status (RBANS) was conducted. Functional annotations were performed using FUMA. Polygenic risk scores (PRSs) for antidepressant non‐remission and symptom improvement were using PRSice v2. Associations between PRSs and cognitive domains were analyzed, adjusting for age, sex, and genomic principal components. Bonferroni correction and permutation tests were applied to address multiple testing issues.

**Result:**

Out of the five cognitive domains, significant SNPs were identified for the attention domain (lead SNP rs67854110, beta = ‐9.91, CI = [‐13.12, ‐6.70], P = 4.4e^−09^). Top suggestive genes associated with language showed differential expression in the hypothalamus, cortex, and nucleus accumbens (P_bon_ = 0.001). The language gene set analysis exhibited significant enrichment with the GWAS Catalog set response to cognitive‐behavioral therapy in depression (P = 9.24e^−11^). Additionally, the top SNP associated with delayed memory (rs13087568, beta = ‐8.24, CI = [‐11.36, ‐5.11], P = 4.5e^−07^) was previously linked to depressive symptoms by other studies. Polygenic risk scores (PRS) for non‐remission negatively correlated with attention (P_Threshold_ = 0.0001, N_SNPS_ = 39, OR = 0.105 [0.016, 0.686], P = 0.019), immediate memory (P_Threshold_ = 0.001, N_SNPS_ = 320, OR = 0.074 [0.011, 0.490], P = 0.0072), and delayed memory (P_Threshold_ = 0.01, N_SNPS_ = 2449, OR = 0.107 [0.021, 0.540], P = 0.0074). PRS for symptom improvement showed a positive correlation with delayed memory (PThreshold = 0.001, N_SNPS_ = 385, OR = 5.329 [1.056, 26.885], P = 0.044). However, none of the PRS associations survived the Bonferroni threshold.

**Conclusion:**

These findings suggest a genetic link between cognitive domains and antidepressant response in LLD, reinforcing previous associations. Understanding genetic contributions to cognitive decline in older adults with depression could aid in early identification and interventions to prevent dementia.

